# Education- and income-based inequalities of functional dentition by dental service utilization

**DOI:** 10.3389/froh.2025.1557008

**Published:** 2025-06-13

**Authors:** Anna Rachel dos Santos Soares, Carlos Antonio Gomes da Cruz, Maria Luíza Viana Fonseca, Líria Sheila Chamane, Loliza Luiz Figueiredo Houri Chalub, Raquel Conceição Ferreira

**Affiliations:** Department of Social and Community Dentistry, School of Dentistry, Universidade Federal de Minas Gerais, Belo Horizonte, Minas Gerais, Brazil

**Keywords:** oral health, healthcare disparities, health inequality monitoring, tooth loss, adults, facilities and services utilization

## Abstract

**Introduction:**

This study analyzed the magnitude of education- and income-based inequalities in functional dentition (FD) among Brazilian adults between 2013 and 2019, considering dental service utilization.

**Methods:**

This study based on repeated cross-sectional surveys using secondary data from a probabilistic sample of adults (18–59 years old) who participated in the 2013 and 2019 National Health Survey (NHS). Only individuals who reported having used dental services were included. FD was defined as having ≥21 teeth, based on self-reported tooth loss in the maxillary and mandibular arches. Variables included sex, age, education (years of study), and per capita family income (measured in minimum wages). Dental service utilization was assessed using the question “When was the last time you visited a dentist?” with responses dichotomized into “>1 year” and “≤1 year”. Absolute and relative inequalities in FD were assessed using the slope index of inequality (SII) and the relative index of inequality (RII), respectively, based on education and income. Generalized linear models (log-binomial regression) were applied with a logarithmic link function to estimate RII (rate ratios) and an identity link function to estimate SII (rate differences), adjusting for sex and age. Trends between 2013 and 2019 and differences in SII and RII by dental service utilization were assessed through two-way interaction terms in the models. All analyses accounted for the survey's complex sampling design and sample weights.

**Results:**

The prevalence of FD was 85.95% (2013) and 89.45% (2019) (*p* < 0.001). SII and RII indicated greater FD prevalence among higher socioeconomic groups, with the magnitude of education-based inequality higher than income-based inequality. Education-based inequalities decreased from 2013–2019. Educational inequalities were more pronounced among those who used dental services >1 year (*p* < 0.001), whereas income-based inequalities did not differ by dental service utilization (*p* > 0.05).

**Conclusions:**

Despite the reduction in FD education-based inequality in Brazil, persistent disparities were observed between socioeconomic groups, with the lowest inequalities found among adults who used dental services ≤1 year. This indicates the importance of interventions aimed at reducing barriers and promoting access to services for the most vulnerable populations.

## 1 Introduction

Functional dentition (FD) has been defined by the World Health Organization (WHO) as a key outcome for global oral health monitoring. Among its 2020 targets, the WHO aimed to increase the number of adults and older individuals with at least 21 natural teeth, without the need for dental prostheses (1). FD refers to a dentition configuration that enables the maintenance of the minimum functions such as chewing, esthetics, and phonetics (2). Ensuring the maintenance of FD should be a priority in equitable public policies, particularly in settings where tooth loss is prevalent, and rehabilitation prosthetics are inaccessible to many, especially for socially disadvantaged individuals. In Brazil, studies have reported a high prevalence of lack of functional dentition among adults (almost 25%) and edentulism in older individuals (53.7%) (3), along with a significant demand for prosthetic rehabilitation (4). Other studies have found a different scenario: the mean number of remaining teeth was 22.3 in adults aged 18 or older in Chile (5), and the prevalence of edentulism was 15.7% among adults aged 20 or older in Colombia (6).

Social determinants of health are associated with FD. Higher FD prevalence has been observed among individuals with greater educational attainment and income, while tooth loss is more common among disadvantaged populations (3, 7–10). Structural social determinants—such as socioeconomic and political contexts, alongside indicators of social position such as income and education—shape social hierarchies and influence access to resources, goods, services, and exposure to health risks (11, 12). Income has both material and psychosocial effects. Materially, it enables access to essential resources, such as healthier food, better housing conditions, materials, and goods for health care. The psychosocial effect of income must be related to symbolic resources, including social prestige. Similarly, education plays a dual role, equipping individuals with knowledge and skills for disease prevention while also enhancing their social standing and employment opportunities (13).

Social and economic indicators have also been recognized as determinants of dental service utilization (14). A literature review revealed a direct association between higher education, increased income, and greater dental service utilization. In higher-income groups with better socioeconomic status, both dental service utilization rates and annual dental visits were higher than in lower-income groups (15). The National Oral Health Policy (Política Nacional de Saúde Bucal—PNSB), implemented in 2004 in Brazil, aims to expand access to oral health services for the Brazilian population, proposing a reshaping of the care model “articulating the individual with the collective, promotion and prevention with treatment and recovery of the population's health”. Based on the PNSB, access to conservative treatment for adults and older adults was expanded, overcoming the model restricted to mutilating treatment in emergency consultations. Based on the principles of universality and equity, the PNSB aims to reduce inequalities in oral health (16). After the implementation of the PNSB, there was an expansion in the coverage of public oral health services in the country. Despite this, universal coverage is still facing challenges. Data from 2019 indicated dental health team coverage of approximately 43% and first dental consultation coverage was only 4.2% with a decreasing trend (17). This expansion has particularly benefited less socially favored groups. A higher proportion of low-income, low-education adults rely exclusively on the Unified Health System (SUS) for oral health care, as observed in the FIOCRUZ oral health indicators panel (18). This situation raises the hypothesis that the utilization of health services may contribute to reducing health inequities by providing disadvantaged groups with better access to preventive and curative care. In this study, regular dental service use was employed as a proxy for access to both preventive and conservative dental care, which may contribute to the maintenance of a higher number of natural permanent teeth. Regular dental visits can thus represent opportunities for preventive care, particularly if the care model prioritizes the preservation of natural teeth (8, 19, 20). Consistent with this hypothesis, previous studies in Brazil have shown that regular dental service utilization can help reduce disparities in edentulism between groups with higher and lower levels of education (8).

Continuous monitoring of oral health inequalities using specific measures to assess their magnitude is essential for evaluating the effectiveness of health policies in Brazil. This study adds to the existing literature by examining the magnitude of socioeconomic inequalities in FD among Brazilian adults changed between 2013 and 2019, considering the dental service utilization The use of both absolute (SII) and relative (RII) measures of inequality strengthens the methodological rigor and enables temporal comparisons. Studies in Brazil (21, 22) and other countries across South (5, 6) and North America (23, 24), Europe (25, 26), Asia (27, 28) and Australia (29) have demonstrated socioeconomic inequalities in oral health outcomes and considering dental service utilization. However, few studies compared inequalities in oral health outcomes based on dental service utilization or evaluated the effect of dental service utilization on the magnitude of inequalities (8, 25, 26). In Brazil, inequalities in functional dentition were evaluated only among elders, comparing data from 2003–2010 without considering dental service utilization (30).

Monitoring efforts are crucial, especially since universality has been identified as a key strategy for reducing persistent oral health inequities globally, as highlighted in the Bangkok Declaration (31). Analyzing the magnitude of FD inequalities across different social groups, while considering dental service utilization, allows for an assessment of the long-term impact of public policies. A reduction in FD inequalities is expected, as increased access to public services has likely provided less socially advantaged groups with greater opportunities for the promotion, prevention, and maintenance of oral health. Therefore, this study aimed to calculate changes in the FD prevalence and compare the magnitude of education- and income-based inequality in FD between 2013 and 2019, as well as to examine differences in this magnitude according to dental service utilization among Brazilian adults.

## 2 Materials and methods

This cross-sectional study analyzed public secondary data from the National Health Survey (NHS) conducted in Brazil in 2013 and 2019. The databases and variable dictionaries were obtained from the Instituto Brasileiro de Geografia e Estatística (IBGE) website in June 2022, with databases updates and applied in August 2020 (NHS 2013) and May 2022 (NHS 2019). These updates included adjustments to sample weights based on population projections by state, sex, and age for the period of 2010–2060 (32). The NHS 2013 (CAAE: 10853812.7.0000.0008) and 2019 (CAAE: 11713319.7.0000.0008) projects were approved by the National Research Ethics Committee, and the participants provided informed consent.

### 2.1 Sample selection and data collection

The study sample comprised residents of permanent private households in both urban and rural areas across Brazil's five macro-regions, states, capitals, and metropolitan regions. Eligibility criteria included individuals aged 18 years or older (2013) or 15 years or older (2019). Sample size calculations considered estimated proportions and their 95% confidence intervals (95% CI), design effect, number of residents per primary sampling unit, and the proportion of households with eligible participants. A three-stage random selection process was employed: census tracts, households, and individuals. In each census tract, 10–14 households were randomly selected based on tract size to meet the required sample size. Within each household, one individual was randomly chosen with equal probability among eligible participants. Data were collected through structured interviews conducted by trained researchers. The questionnaire included items on household characteristics and demographic and socioeconomic attributes of both residents and the selected respondents. Further methodological details are available in a prior publication (33). In this study, data from participants aged 18–59 years were analyzed. Individuals aged 60 years or older were excluded, as FD is associated with tooth loss—a condition linked to aging and more prevalent among older adults.

### 2.2 Variables

The oral health outcome, FD, was defined based on the number of present teeth and assessed through self-reported tooth loss in the maxillary and mandibular arches, which represents a validated method for assessing tooth loss in adult populations (34) that has been used in other studies (25, 26). The total number of natural teeth was calculated by subtracting the number of missing teeth from 32, which represents a complete permanent dentition without tooth loss. FD was then classified as follows: without FD (0–20 natural teeth) and with FD (≥21 natural teeth).

Socioeconomic variables included education and per capita income. Education was measured in years of study based on the highest level of schooling completed, following a classification used in previous studies: 0–4; 5–8; 9–11; and ≥12 years of study (3, 7, 8). Per capita income was calculated as the total household income divided by the number of residents and converted into minimum wages (MW) (2013: BRL 678.00—USD 332.00 and 2019: BRL 998.00—USD 261.00), a calculation method established in the literature (19). It was then categorized into four groups, as was done previously (35): 0–1 MW; 1.1–2 MW; 2.1–3 MW, and ≥3.1 MW.

The covariates included dental services utilization, sex (male, female), and age. Dental services utilization was assessed through the question “When was the last time you visited a dentist?”, with the following response options: never visited a dentist; visited a dentist in the last year; between 1 and less than 2 years; between 2 and less than 3 years; and 3 or more years ago. In this study, this variable was categorized as: used dental services within the last year and used dental services more than a year ago. Responses from individuals who had never used dental services were excluded to enable comparisons between different frequencies of service utilization (more vs. less regular). Additionally, FD is influenced by prior tooth loss, which typically occurs within the context of dental care. Age was recorded in complete years and categorized into the following groups as previous studies (35, 36): 18–24 years, 25–39 years, and 40–59 years (13). The WHO social determinants of health model, recognized as appropriate for studies on oral health inequalities (12), provided the conceptual framework for selecting the outcome, socioeconomic variables, and covariates.

### 2.3 Data analysis

Data were subjected to descriptive analysis to characterize the sample and obtain the prevalence of FD. A logistic regression model was used to assess associations between FD and education, income, and dental service utilization for 2013 and 2019, as well as interactions between socioeconomic indicators and dental service utilization. Marginal estimates were computed to obtain the predicted probability of FD according to levels of education, income, and dental service utilization, adjusted for sex and age, with results represented graphically. The observed change in the FD prevalence between surveys was calculated (Δ2013_2019). The significance of this change was tested by hypothesis testing using the Student-t distribution, dividing Δ by the standard error of the change and estimating the confidence interval of the change at a 95% significance level, rejecting the null hypothesis when the confidence interval did not include zero (37).

The magnitude of absolute and relative education and income-based inequalities of FD was assessed using the Slope Index of Inequality (SII) and Relative Index of Inequality (RII), both adjusted for sex and age (38). These regression-based indices account for the full socioeconomic distribution rather than comparing only the most extreme groups. The indices are obtained by including a *ridit-*score in the regression model, calculated from the ranking of social groups defined by education and income, from lowest to highest. The *ridit*-score is based on the midpoint of the cumulative distribution interval of participants in a given category (38). The RII and SII were estimated using generalized linear regression models—log-binomial regression with a logarithmic link function for RII and an identity function for SII (39). When the model did not converge using a binomial distribution, the Poisson family was used instead. Both indices were estimated with 95% confidence intervals (CI). The RII can be interpreted as the prevalence ratio (PR), while the SII represents the absolute difference in prevalence between the highest and lowest positions in the distribution of education and income indicators. The RII assumes values greater than one when FD is more prevalent among advantaged individuals and values less than one when FD is more frequent among disadvantaged groups. If no inequality exists, RII equals one, with greater deviations indicating stronger inequality. Similarly, higher absolute SII values indicate greater inequality, with positive values signifying higher FD prevalence in advantaged groups and negative values indicating greater FD prevalence among disadvantaged individuals. If no disparity is present, SII equals zero.

Trends in adjusted RII and SII over time were examined by including a two-way interaction term between *ridit*-scores and survey year (NHS 2013 and 2019). A statistically significant interaction coefficient indicated changes in adjusted RII and SII over time. Additionally, the SII and RII were estimated separately for each subpopulation based on dental service utilization: among adults who had used dental services either within the last year or more than a year ago. To estimate differences in RII and SII according to dental service utilization within each survey, a two-way interaction term between the *ridit*-score and dental service utilization (ridit_use = ridit × dental service utilization) was included in the model. For these models, in each subpopulation, the trend in RII and SII over time was also assessed. The *lincom* command in Stata® was performed for the total sample and considering subpopulations defined by education, income, and dental service utilization. All analyses were performed using the Stata® program, version 18.0 (StataCorp LP, College Station, TX, USA), accounting for the complex sampling design and sampling weights.

## 3 Results

**Figure 1:**
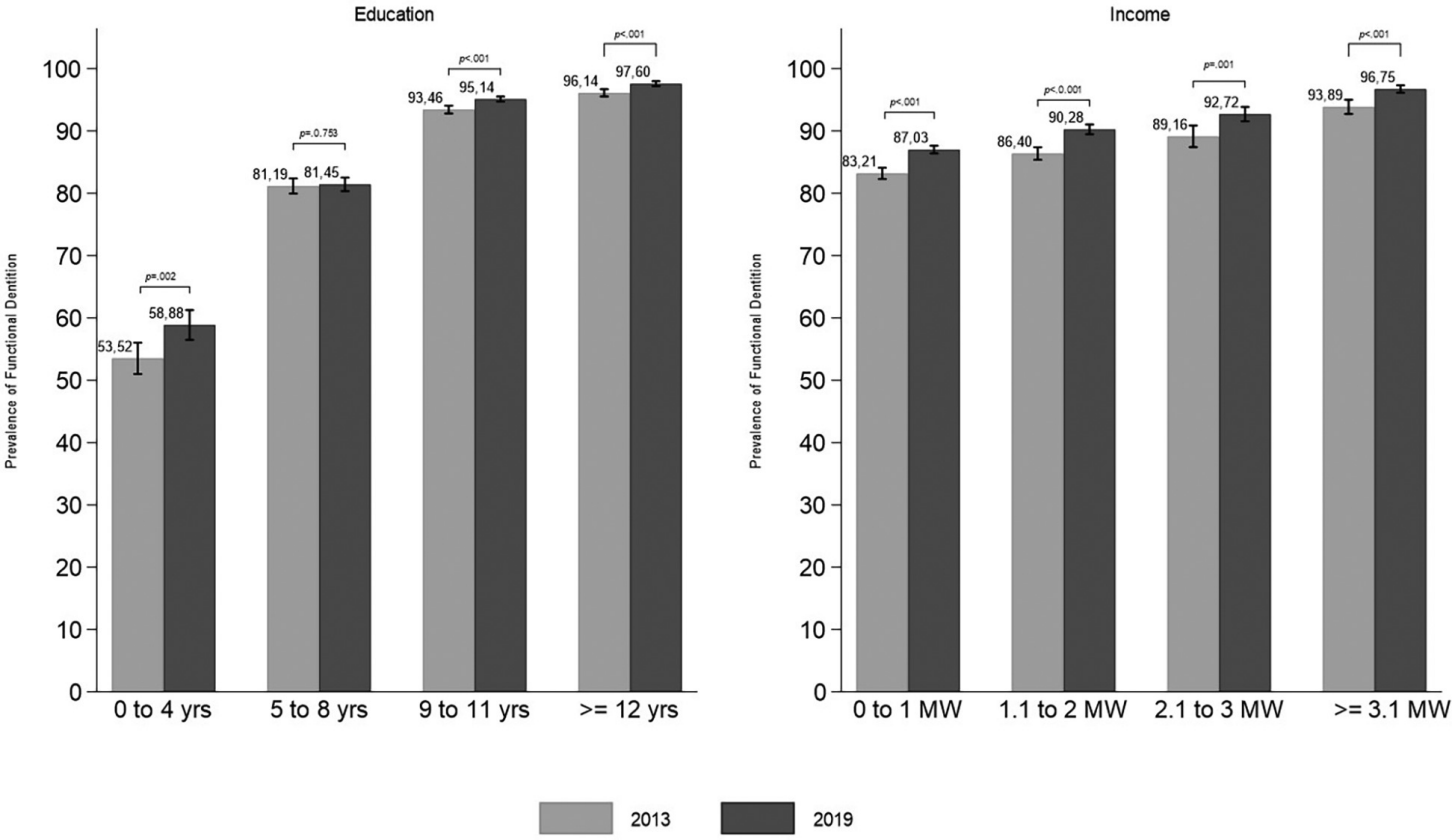
Prevalence of functional dentition by education and income levels among Brazilian adults in 2013 and 2019, with *p*-values for differences between surveys (NHS 2013 and NHS 2019).

Of the 60,308 participants in 2013 and 90,846 in 2019, a total of 47,491 (78.75%) and 64,318 (70.80%), respectively, met the inclusion criteria of being 18–59 years old, having a response for the self-reported tooth loss in the maxillary and mandibular arches and having used dental services. In the two surveys, the majority of the total sample was female and aged 40–59. In both 2013 and 2019, adults with fewer years of study (0–4) and lower income (0–1 MW) had the lowest prevalence of FD, while individuals who used dental services within the last year had a higher prevalence of FD (Table 1). The overall prevalence of FD increased from 85.95% in 2013 (*n* = 8,534; 95% CI: 85.34–86.54) to 89.45% in 2019 (*n* = 8,902; 95% CI: 89.02–89.87) (Δ2013_2019: 3.50 95% CI: 2.74; 4.26; *p* < 0.001). In both years, FD was more prevalent among individuals with the highest education level (≥12 years of study) and the highest income (≥3.1 MW). The largest change in FD prevalence (Δ2013–2019) was observed among adults with fewer years of study (0–4); however, significant changes were also observed in the two higher education levels (9–11 and ≥12 years of study) (Figure 1). The differences in the prevalence of FD across income levels were significant, and the Δ2013–2019 value was similar for all income levels.

**Table 1:** Sample structure and prevalence of functional dentition (FD) by demographic and socioeconomic characteristics and dental service utilization in Brazil (NHS 2013 and 2019).

Variables	2013				2019			
	Total sample (*n* = 47,491)		With FD (*n* = 40,583)		Total sample (*n* = 64,318)		With FD (*n* = 55,959)	
	*n*	w% (95% CI)	*n*	w% FD (95% CI)	*n*	w% (95% CI)	*n*	w% FD (95% CI)
Demographic characteristics								
Sex								
Male	20,446	47.40 (46.57; 48.24)	17,938	88.14 (87.23; 89.00)	30,472	47,43 (46,74; 48,12)	27,148	91.45 (90.88; 91.99)
Female	27,045	52.60 (51.76; 53.43)	22,645	83.97 (83.19; 84.72)	33,843	52,57 (51,88; 53,26)	28,811	87.65 (86.97; 88.30)
Age groups								
18–24 years old	7,471	19.22 (18.55; 19.91)	7,460	99.88 (99.67; 99.96)	7,844	17.48 (16.83; 18.15)	7,820	99.74 (99.53; 99.85)
25–39 years old	20,183	38,85 (38.06; 39.65)	19,461	96.86 (96.46; 97.22)	24,828	37.30 (36.62; 37.98)	24,287	98.27 (98.03; 98.49)
40–59 years old	19,837	41.92 (41.13; 42.73)	13,662	69.45 (68.18; 70.69)	31,646	45.23 (44.50; 45.95)	23,852	78.20 (77.34; 79.04)
Socioeconomic characteristics								
Education (years of study)								
0–4	5,981	12.01 (11.47; 12.58)	3,312	53.52 (51.03; 56.00)	6,420	7.96 (7.59; 8.34)	3,642	58.88 (56.50; 61.23)
5–8	12,813	26.83 (26.03; 27.65)	10,347	81.19 (79.94; 82.38)	17,410	25.09 (24.43; 25.76)	13,914	81.45 (80.34; 82.51)
9–11	18,174	39.50 (38.69; 40.33)	16,858	93.46 (92.80; 94.06)	24,547	41.26 (40.55; 41,98)	22,929	95.14 (94.72; 95.52)
≥12	10,523	21.66 (20.77; 22.57)	10,066	96.14 (95.51; 96.68)	15,941	25.69 (24.84; 26.56)	15,474	97.60 (97.18; 97.97)
Income (in minimum wages)								
0–1	25,436	50.09 (49.07; 51.11)	21,074	83.21 (82.30; 84.08)	36,742	53.23 (52.33; 54.14)	31,003	87.03 (86.41; 87.63)
1.1–2	12,029	28.73 (27.93; 29.53)	10,280	86.40 (85.37; 87.37)	15,393	27.47 (26.77; 28.18)	13,591	90.28 (89.48; 91.03)
2.1–3	4,180	9.31 (8.82; 9.83)	3,740	89.16 (87.32; 90.76)	5,207	8.73 (8.33; 9.15)	4,720	92.72 (91.49; 93.79)
≥3.1	5,836	11.87 (11.16; 12.63)	5,480	93.89 (92.64; 94.93)	6,957	10.57 (9.95; 11.22)	6,626	96.75 (96.09; 97.29)
Dental service utilization (time since the last dental visit)								
>1 year	24,823	50.87 (49.97; 51.77)	19,915	80.76 (79.80; 81.68)	31,053	45,98 (45,25; 46,71)	25,505	85.47 (84.80; 86.12)
≤1 year	22,668	49.13 (48.23; 50.03)	20,668	91.33 (90.65; 91.95)	33,265	54,02 (53,29; 54,75)	30,454	92.84 (92.37; 93.28)

Analyses accounted for the effects of sample design and weighting. w%, weighted percentage.

**Figure 2:**
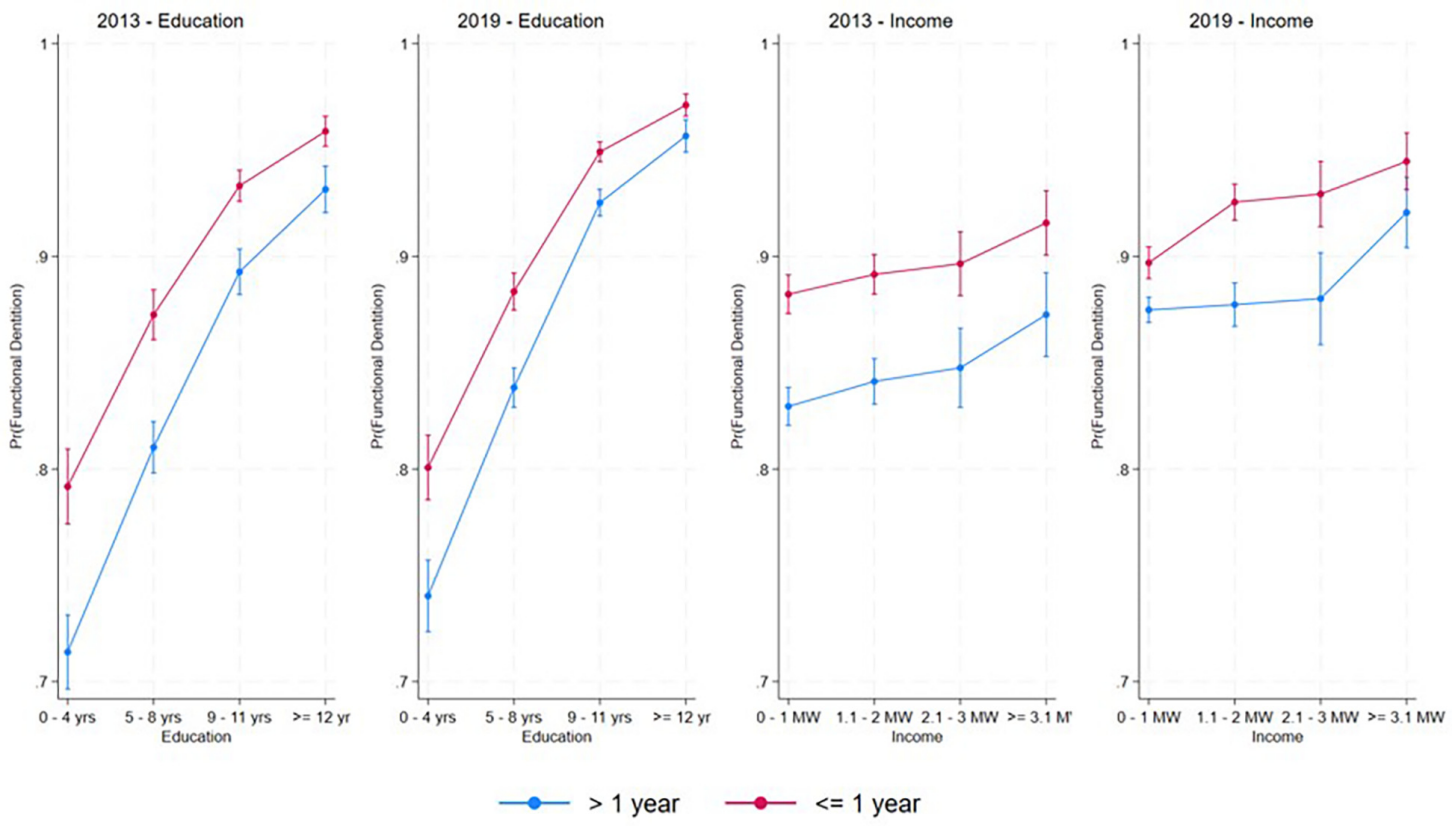
Marginal estimates of predicted probability (Pr) of functional dentition according to levels of education, income, and dental service utilization, adjusted for sex and age.

Higher education, higher income, and dental service utilization within the last year were associated with an increased likelihood of FD in both 2013 and 2019 (Table 2). In 2013, individuals who used dental services within the last year exhibited a higher probability of FD, irrespective of their income or education level (Table 2, Figure 2). In 2019, a similar result was observed regarding education, with a higher probability of FD among individuals who had used dental services within the last year, independent of their education level. Adults with a higher education level had a higher probability of FD in both years, regardless of the time since their dental service utilization (Figure 2). A significant interaction between income and dental service utilization was observed in 2019 (Table 2). In both years, a higher probability of FD was found among adults who used dental services within the last year, compared to those who had used dental services more than a year ago within the same education and income range. These differences were only significant for income levels between 0 and 3 MW (0–1 MW + 1.1–2 MW + 2.1–3 MW) in 2019 (Figure 2). Still in 2019, among adults who used dental services within the last year, those with the lowest income (0–1 MW) had a lower probability of FD than those with higher income levels (1.1–2, 2.1–3, and ≥3.1 MW), despite using dental services with the same frequency. For adults in 2019 who had used dental services more than a year ago, the probability of FD was similar across all three income categories up to 3 MW. In this same year, adults with an income greater than 3.1 MW showed a similar probability of FD, regardless of the frequency of dental service utilization (Figure 2).

**Table 2:** Logistic regression model for the association among functional dentition and sex, age group, education, income, and dental service utilization in Brazil (NHS 2013 and 2019).

Variables	2013		2019	
	OR (95% CI)	*p*-value	OR (95% CI)	*p*-value
Demographic characteristics				
Sex				
Male	1		1	
Female	0.63 (0.56; 0.70)	<0.001	0.58 (0.53; 0.64)	<0.001
Age groups				
18–24 years old	1		1	
25–39 years old	0.04 (0.02; 0.11)	<0.001	0.16 (0.09; 0.30)	<0.001
40–59 years old	0.004 (0.001; 0.01)	<0.001	0.01 (0.01; 0.00)	<0.001
Socioeconomic characteristics				
Education (years of study)				
0–4	1		1	
5–8	2.06 (1.79–2.37)	<0.001	2.15 (1.88; 2.46)	<0.001
9–11	4.58 (3.93–5.34)	<0.001	5.81 (5.07; 6.66)	<0.001
≥12	7.90 (6.36–9.80)	<0.001	10.77 (8.59; 13.49)	<0.001
Income (in minimum wages)				
0–1	1		1	
1.1–2	1.12 (0.99; 1.27)	0.064	1.03 (0.90; 1.18)	0.680
2.1–3	1.20 (0.98; 1.47)	0.075	1.07 (0.81; 1.40)	0.650
≥3.1	1.58 (1.24; 2.01)	<0.001	1.88 (1.41; 2.49)	<0.001
Dental service utilization (time since the last dental visit)				
>1 year	1		1	
≤1 year	1.77 (1.58; 1.99)	<0.001	1.32 (1.41; 2.49)	<0.001
Interaction between income and the dental service utilization*^,^**				
1.1–2 # ≤ 1 year	-	-	1.50 (1.21; 1.85)	<0.001
2.1–3 # ≤ 1 year	-	-	1.54 (1.04; 2.30)	0.033
≥3.1 # ≤ 1 year	-	-	1.19 (0.81; 1.74)	0.379

Analyses accounted for the effects of sample design and weighting.

* The interactions between education and dental service utilization, as well as between income and dental service utilization, were not significant in 2013. For that, the interaction terms were not included in the 2013 regression model.

** In 2019, there was no significant interaction between education and dental service utilization.

**Figure 3:**
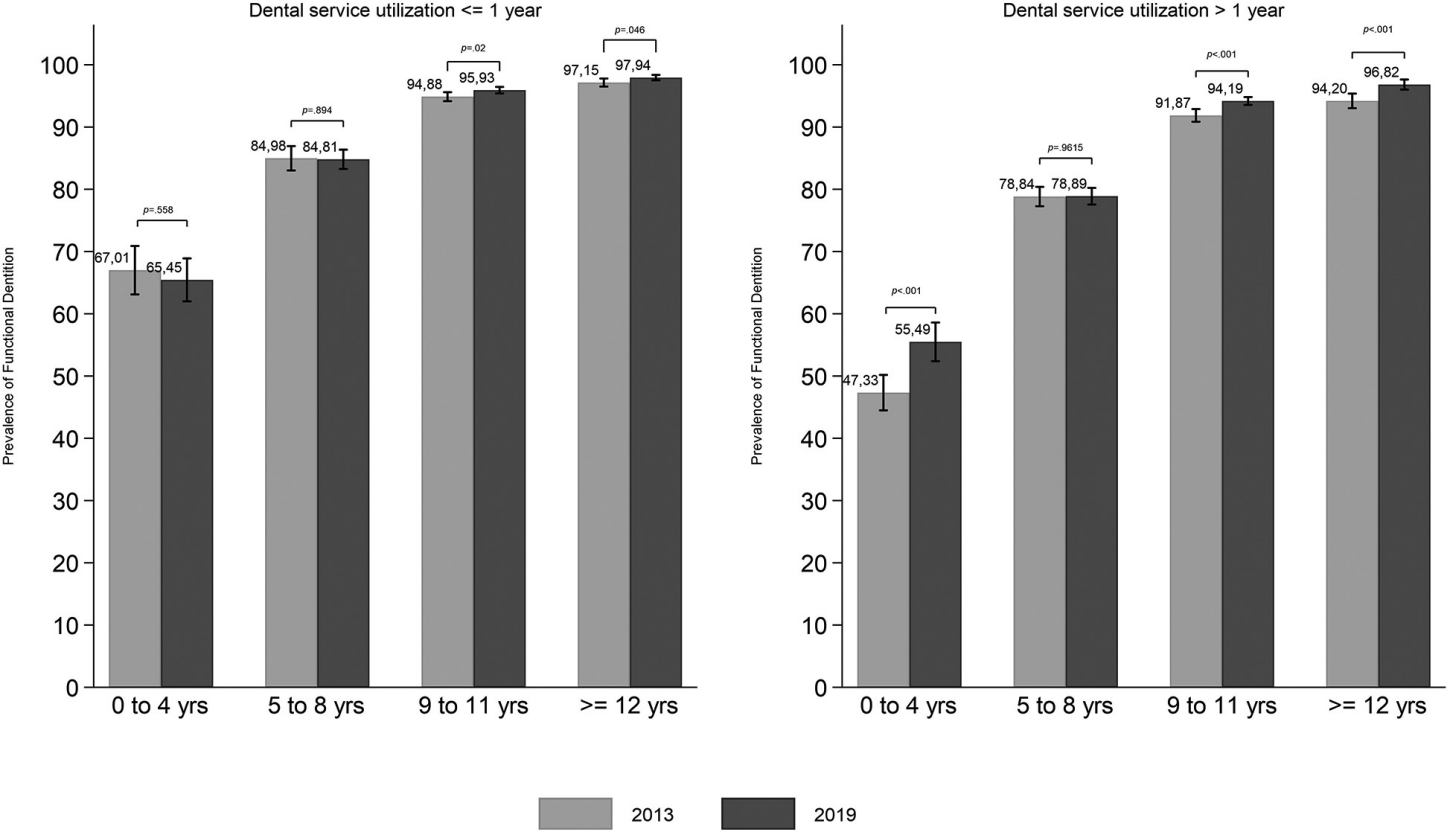
Prevalence of functional dentition by education among Brazilian adults in 2013 and 2019 according to dental service utilization (time since the last dental visit), with *p*-values for differences between survey years.

**Figure 4:**
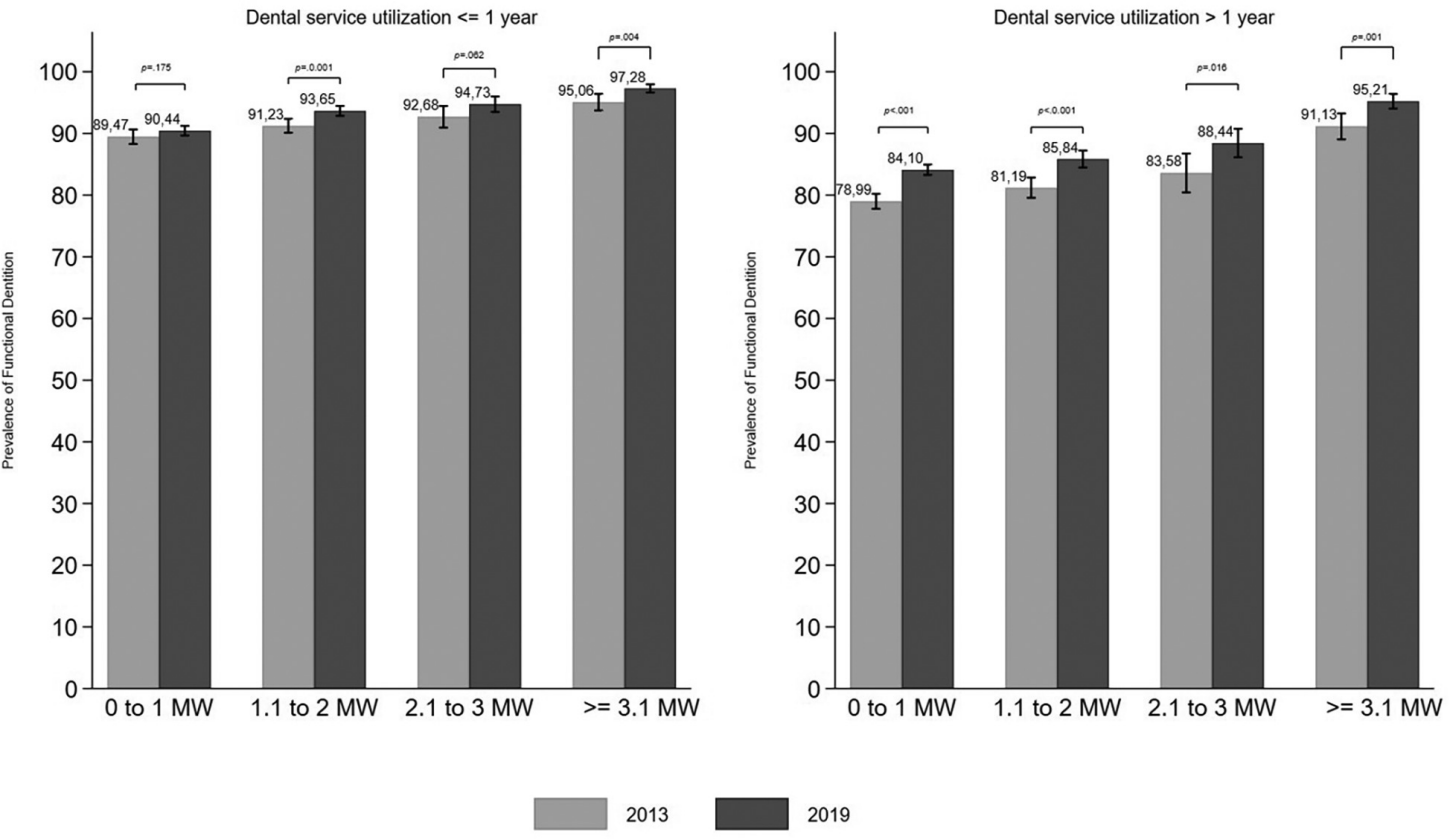
Prevalence of functional dentition by income among Brazilian adults in 2013 and 2019 according to dental service utilization (time since the last dental visit), with *p*-values for differences between survey years.

The comparison of FD prevalence between 2013 and 2019 for each education and income level according to dental service utilization was shown in Figures 3, 4. The lowest prevalence of FD was observed for those with 0–4 years of study and who used dental services more than one year ago. The highest increase in the FD prevalence between 2013 and 2019 was observed in this group (*p* < 0.05). Significant changes in the FD prevalence between the two surveys were also observed in the two higher education levels, regardless of the time since dental service utilization (Figure 3). Despite the lower FD prevalence among adults who last used dental services more than one year ago, all income levels experienced a significant increase in prevalence between 2013 and 2019 (Figure 4).

For the total sample in the two surveys, the SII values were positive, and the RII values exceeded one, indicating a higher prevalence of FD among the most advantaged groups in terms of both education and income. Education inequality exhibited a greater magnitude than income-based inequality, as evidenced by higher SII and RII values for education compared to income. Both SII and RII for education declined from 2013–2019. The statistically significant interaction term (*ridit-score* × survey year), with a negative value (SII) and below one (RII), confirmed this downward trend. In contrast, no significant decrease was observed in absolute (SII) or relative (RII) income-based inequality between 2013 and 2019 (Table 3).

**Table 3:** Slope index of inequality (SII) and relative index of inequality (RII) with 95% confidence intervals (CI) for education- and income-based inequalities in functional dentition among Brazilian adults in 2013 and 2019 for total sample and subpopulations stratified by dental service utilization, with *p*-values for trends in inequality magnitude over time and differences in RII and SII by dental service utilization.

Education-based inequality	2013	2019	Term of interaction *ridit-score # survey year*	*p*-value for trend
Total sample				
SII (95% CI)	0.39 (0.36; 0.42)	0.35 (0.33; 0.37)	−0.0780 (−0.1144; −0.0416)	**<0**.**001**
RII (95% CI)	1.52 (1.47; 1.56)	1.43 (1.40; 1.46)	0.9006 (0.8658; 0.9367)	**<0**.**001**
Subpopulation analysis				
SII (95% CI) Adults who used dental services in the last year	0.26 (0.22; 0.32)	0.26 (0.25; 0.33)	−0.0234 (−0.0679; 0.0211)	0.303
SII (95% CI) Adults who used dental services >1 year	**0.44** (**0.35; 0.46)**	**0.40** (**0.37; 0.43)**	**−0.0870** (**−0.1393; −0.0346)**	**0**.**001**
*p*-value for differences in the SII according to dental service utilization	**<0.001**	**<0.001**	-	
RII (95% CI) Adults who used dental services in the last year	1.32 (1.25; 1.41)	1.30 (1.28; 1.41)	0.967 (0.9230; 1.0133)	0.160
RII (95% CI) Adults who used dental services >1 year	**1.61** (**1.46; 1.63)**	**1.51** (**1.47; 1.56)**	**0.8769** (**0.8275; 0.9293)**	**<0**.**001**
*p*-value for differences in the RII according to dental service utilization	**<0.001**	**<0.001**	-	
Income-based inequality	2013	2019	Term of interaction *ridit-score # survey year*	*p*-value for trend
Total sample				
SII (95% CI)	0.18 (0.16; 0.21)	0.17 (0.16; 0.19)	0.0069 (−0.0181; 0.0320)	0.589
RII (95% CI)	1.21 (1.18; 1.23)	1.19 (1.17; 1.20)	0.9999 (0.9751; 1.0253)	0.994
Subpopulation analysis				
SII (95% CI) Adults who used dental services in the last year	0.13 (0.10; 0.19)	0.13 (0.11; 0.18)	0.0180 (−0.011; 0.0472)	0.226
SII (95% CI) Adults who used dental services >1 year	0.16 (0.13; 0.22)	0.18 (0.15; 0.20)	0.0274 (−0.0158; 0.0706)	0.213
Differences in the SII according to dental service utilization	0.657	0.099	-	
RII (95% CI) Adults who used dental services in the last year	1.18 (1.14; 1.28)	1.19 (1.16; 1.22)	1.0218 (0.9780; 1.0677)	0.335
RII (95% CI) Adults who used dental services >1 year	1.14 (1.10; 1.21)	1.13 (1.11; 1.20)	1.0148 (0.986; 1.0445)	0.317
Differences in the RII according to dental service utilization	0.443	**0.052**	-	

Analyses accounted for the effects of sample design and weighting.

* The SII and RII were adjusted by sex and age groups. Bold values indicate statistically significant trends (*p* < 0.05). Bold values indicate statistically significant differences in SII or RII according to dental service utilization (*p* < 0.05).

Among adults who used dental services within the last year or more than a year ago, the SII was positive and the RII was greater than one in both 2013 and 2019, indicating persistent absolute and relative inequalities in FD based on education and income, favoring individuals with higher socioeconomic status (Table 3). Regarding education-based inequality among adults who had used dental services more than a year ago, the SII was nearly twice the value observed among those who used dental services within the last year, in both 2013 and 2019. The significant interaction term between education and dental service utilization (*p* < 0.001 in both years) indicates that absolute (SII) and relative (RII) inequalities were significantly higher among individuals who used dental services more than a year ago. Regarding income-based inequality, there were no significant differences in SII and RII between those who used dental services in the last year and those who did not, as evidenced by the non-significant interaction term between *ridit*-score and dental service utilization (*p* > 0.05) (subpopulation analysis Table 3).

## 4 Discussion

The findings of this study indicate an increase in the prevalence of FD among Brazilian adults from 2013–2019, while also highlighting persistent social inequalities, particularly concerning education levels. Individuals with higher education and income consistently demonstrated a higher prevalence of FD. The study also demonstrated a higher magnitude of education-based inequality compared to the magnitude of income-based inequality and a decrease in educational inequalities was observed between 2013 and 2019. A greater magnitude of educational inequality was observed among those who used dental services more than one year ago.

In contrast, those who had used dental services within the past year showed a higher prevalence of FD. This result may reflect increased access to preventive and conservative treatments over the lifetime. Previous study has shown that among individuals who have visited a dentist, edentulous people were more likely to have had their last visit over 12 months ago (40). This might be due to longer intervals between appointments leading to the need for more invasive treatments like extractions. In contrast, regular use of dental services could be associated with more conservative treatments that focus on prevention and health promotion, which can help reduce the need for invasive interventions (8). This finding may also indicate a shift in oral health care models, with greater emphasis on prevention and promotion, potentially helping to preserve natural teeth in adulthood among Brazilians.

Considering the total sample, the lowest prevalence of FD (i.e., the poorest oral health status), was observed among those with the lowest education levels in both years. In both surveys, approximately 70% of individuals with low education levels also reported low income. Among participants with the lowest income, 16.18% in 2013 and 10.87% in 2019 had the lowest levels of education (data not shown). These findings suggest that adults with lower education levels often face compounded challenges related to low income, which may exacerbate their social disadvantage (7–10, 12, 13, 30). Education influences not only health-related knowledge, skills, and behaviors but also access to resources and services (11, 13). As a result, it can mitigate some of the negative effects of low income on health. Individuals with higher levels of education are generally more likely to adopt healthy behaviors, understand healthcare advice, and make informed decisions about their well-being (13). Furthermore, education is associated with greater ability to navigate the healthcare system, better access to preventive and therapeutic information, and increased awareness of health risk factors. Education may also reflect the long-term effects of childhood circumstances on health (13, 30), making it a more appropriate indicator for cumulative life-course measures of disease and treatment—such as the number of teeth, which relates directly to FD. The results underscore the significant role of education in shaping oral health outcomes, even in low-income contexts, and this could be a new contribution to the understanding of oral health inequalities.

Between 2013 and 2019, both absolute and relative reductions in educational-based inequalities regarding FD were observed. This improvement appears to be driven by a significant increase in the prevalence of FD among individuals with the lowest education levels (0–4 years of study) in 2019, compared to 2013, which helped reduce the gap between educational levels. These findings suggest that public policies and collective preventive measures implemented over previous decades (17, 20)—such as water fluoridation and the widespread use of fluoride toothpaste—may have begun to show more pronounced effects among recent adult cohorts, including those from socially disadvantaged backgrounds. The 2019 sample included a higher proportion of individuals born after the 1980s and 1990s, a period characterized by the expansion of these preventive strategies. In contrast, the 2013 sample comprised more individuals born between 1954 and 1995, who had more limited exposure to such measures during childhood. This generational shift may help explain the improved maintenance of natural teeth and the higher prevalence of FD observed in 2019. A similar trend of reduced tooth loss among adults had already been observed in Brazil in 2010 compared to 2003 (3), and was also attributed to improvements in the healthcare system, including increased exposure to water fluoridation and the mass use of fluoride toothpaste. These two interventions reached broad population coverage during the 1980s and 1990s (17) and are considered key factors in the decline in both the prevalence and severity of dental caries—the leading cause of tooth loss in Brazil (3). Additionally, the expansion of public health services and the shift in dental care models—from a predominantly extraction-based approach to more conservative and preventive practices—may have played a role in reducing tooth loss among the adult population. The reorientation of dental practice in Brazil, with an emphasis on promotional, preventive, and conservative actions through the National Oral Health Policy (implemented in 2004) (16), may have contributed to a reduction in extractions and the preservation of teeth affected by caries, especially in the most disadvantaged groups, who are the most frequent users of public oral health services. Studies examining procedures offered by SUS have consistently observed a reduction in extraction rates compared to other procedures performed in primary care between 1998 and 2012 (41), and again from 2008–2018 (42). All of these could explain the increase in FD prevalence from 2013–2019, aligning with global improvements and the WHO targets for oral health in adult and elderly populations (1). However, persistent inequalities were observed, with greater absolute and relative magnitudes when measured according to adults' education levels. This finding is consistent with the stronger association between education and FD observed in the logistic regression model, compared to the association between FD and income.

Consistently across both surveys, the logistic regression model showed a significant association between FD and income only among individuals with the highest income. In 2019, dental service utilization modified the association between income and FD. Among those who had visited a dentist within the last year, individuals with the lowest income (0–1 MW) had the lowest FD prevalence, suggesting that, despite similar patterns of service use, they may face additional barriers to maintaining oral health. Furthermore, the similar FD prevalence observed among adults with income up to 3 MW who had used dental services less frequently (>1 year since the last visit) suggests that other social determinants, such as education, health literacy, and early-life conditions, play a significant role in shaping long-term oral health outcomes. In contrast, individuals with higher incomes (≥3.1 MW) demonstrated similar FD prevalence regardless of their dental service utilization frequency, indicating that the influence of income on health extends beyond healthcare utilization. Material factors, such as housing conditions, access to healthy foods, and availability of essential resources (e.g., hygiene products and a nutritious diet), may also contribute to better oral health outcomes among higher-income groups (12, 13). These determinants are linked to the prevalence and severity of dental caries and periodontal disease—the primary conditions leading to tooth loss—which may explain the higher prevalence of FD in individuals with greater financial resources (3, 7).

When examining inequalities separately among individuals who used dental services within the last year or more than one year ago, significant differences in education-based inequalities indexes were observed, with these inequalities being greater among those who had used dental services a longer time ago. Regular use of dental services may represent an opportunity for access to preventive treatments (8). It is believed that the expansion of access to public services in Brazil may help explain this trend, benefiting those in more disadvantaged social conditions (17). The results of the NHS research indicated that public services were most frequently used by adults with lower education and income who were regular users of dental services (18). In 2013, 48.57% of adults with low education used public dental services in the last year, compared to 9.23% of those with more than 12 years of education. In 2019, these percentages were 52.03% and 9.98%, respectively (data not shown). In 2019, 42.00% of adults with only primary education used public dental services (SUS), compared to 6.2% among those with higher education (data not shown). Additionally, more than half (51.5%) of adults with an income of up to one minimum wage used public dental services, in contrast to only 3.4% among those earning more than three minimum wages (18). Consistent with this finding, a reduction in inequalities in the use of dental services among Brazilians with higher and lower incomes was observed between 1998 and 2008 (43) and related to economic position (22). In this sense, this result may indicate a positive effect of the PNSB in reducing oral health inequalities. Expanding access to oral health services to the Brazilian population, while adhering to the principle of universality and equity, should continue to guide the actions of the PNSB. This evidence is also supported by studies showing that dental service utilization partially explains the inequalities in edentulism (8), number of natural teeth (26) and functional dentition (25).

On the other hand, a previous study conducted among adults in England, Wales, and Northern Ireland found that lower socioeconomic position (a latent variable derived from income, occupational social class, and household income) was directly associated with a lower number of natural teeth. The effect of socioeconomic position on the number of teeth was primarily direct (84%), indicating that it did not operate mainly through behavioral factors (such as smoking and oral hygiene) or dental service utilization. The indirect pathways—through behaviors and dental attendance—played only a modest role in explaining inequalities in tooth retention. According the authors, the strong direct effect of socioeconomic position may reflect the cumulative impact of social disadvantage throughout the life course, which aligns with the nature of tooth loss as a cumulative measure of oral health (44). This evidence is also supported by the findings of the present study, which revealed persistent inequalities even among users of dental services. When analyzing changes in inequality measures over time, stratified by dental service utilization, it was observed that improvements occurred primarily among those who had not used dental services in the last year. Despite this reduction, in 2019, this group still exhibited greater inequality compared to individuals who had used services within the last year. The most substantial increase in the prevalence of FD between 2013 and 2019 was observed among adults with 0–4 years of study. Similarly, other studies reported a significant increase in the prevalence of FD among people with lower education levels between national surveys (5, 30).

Thus, ensuring collective health initiatives that promote intersectoral integration—enabling access to education, goods, and resources necessary for a healthier life—is a crucial strategy for reducing inequities (11, 13). These findings reinforce the idea that, beyond dental service utilization, other social determinants and the positive effects of broad, preventive public health measures may be contributing to improvements in oral health among Brazilians. The findings underscore the need to prioritize health promotion and preventive actions aimed at maintaining natural teeth through oral health policies. The importance of interventions aimed at reducing barriers and promoting access to services for the most vulnerable populations is highlighted. These policies should address not only disparities in the use of services but also the underlying material and social conditions that perpetuate inequities in oral health. These approaches can contribute to social justice and equity (11, 12), in line with the principles of SUS, in the field of health care (16, 20, 22).

The findings should be interpreted with consideration that they were obtained from a subsample of adults (33) who reported having used oral health services, given the outcome analyzed. Those who had never used services were excluded, and this group may represent a more socially disadvantaged situation, which could affect the generalizability of the results. Additionally, participants aged 60 or older were excluded from the sample, as FD is an age-related outcome due to its direct association with tooth loss. This choice may have compromised the precision of the point estimates compared to those obtained from the full sample. The outcome was defined based on self-reports of lost teeth, consistent with previous studies (25, 26). The validity of this method has been previously confirmed concerning information from epidemiological examinations (34). However, this reliance on self-reports may introduce measurement bias. Additionally, there is a potential for recall bias regarding the reported use of dental services, which could influence the observed associations. Finally, it is important to clarify that the data are not longitudinal, since participants from 2013–2019 are not the same and the observed changes do not reflect modifications within the same people over time.

## 5 Conclusion

The prevalence of functional dentition increased from 2013–2019, being higher among adults with higher education, income, and those who used dental services within the last year. The significant increase in the prevalence of FD among individuals with the lowest education level between 2013 and 2019 may have helped reduce the gap between educational levels. Educational inequalities were more pronounced than income-based inequalities, with significant disparities, particularly among those who used dental services more than one year ago.

## Data Availability

Publicly available datasets were analyzed in this study. This data can be found here: [https://www.pns.icict.fiocruz.br/bases-de-dados/], Fiocruz repository.
